# Horizontal transfer of code fragments between protocells can explain the origins of the genetic code without vertical descent

**DOI:** 10.1038/s41598-018-21973-y

**Published:** 2018-02-23

**Authors:** Tom Froese, Jorge I. Campos, Kosuke Fujishima, Daisuke Kiga, Nathaniel Virgo

**Affiliations:** 1 Institute for Applied Mathematics and Systems Research (IIMAS), National Autonomous University of Mexico (UNAM), Mexico City, 04510 Mexico; 2 Center for the Sciences of Complexity (C3), National Autonomous University of Mexico (UNAM), Mexico City, 04510 Mexico; 3 Faculty of Higher Education Aragon, National Autonomous University of Mexico (UNAM), Nezahualcoyotl City, State of Mexico 57130 Mexico; 4 Earth-Life Science Institute, Tokyo Institute of Technology, Meguro-ku, Tokyo 152-8550 Japan; 5 Institute for Advanced Biosciences, Keio University, Tsuruoka, 9970035 Japan; 6 Faculty of Science and Engineering, School of Advanced Science and Engineering, Waseda University, Shinjuku, Tokyo 169-8555 Japan

## Abstract

Theories of the origin of the genetic code typically appeal to natural selection and/or mutation of hereditable traits to explain its regularities and error robustness, yet the present translation system presupposes high-fidelity replication. Woese’s solution to this bootstrapping problem was to assume that code optimization had played a key role in reducing the effect of errors caused by the early translation system. He further conjectured that initially evolution was dominated by horizontal exchange of cellular components among loosely organized protocells (“progenotes”), rather than by vertical transmission of genes. Here we simulated such communal evolution based on horizontal transfer of code fragments, possibly involving pairs of tRNAs and their cognate aminoacyl tRNA synthetases or a precursor tRNA ribozyme capable of catalysing its own aminoacylation, by using an iterated learning model. This is the first model to confirm Woese’s conjecture that regularity, optimality, and (near) universality could have emerged via horizontal interactions alone.

## Introduction

**Figure 1:**
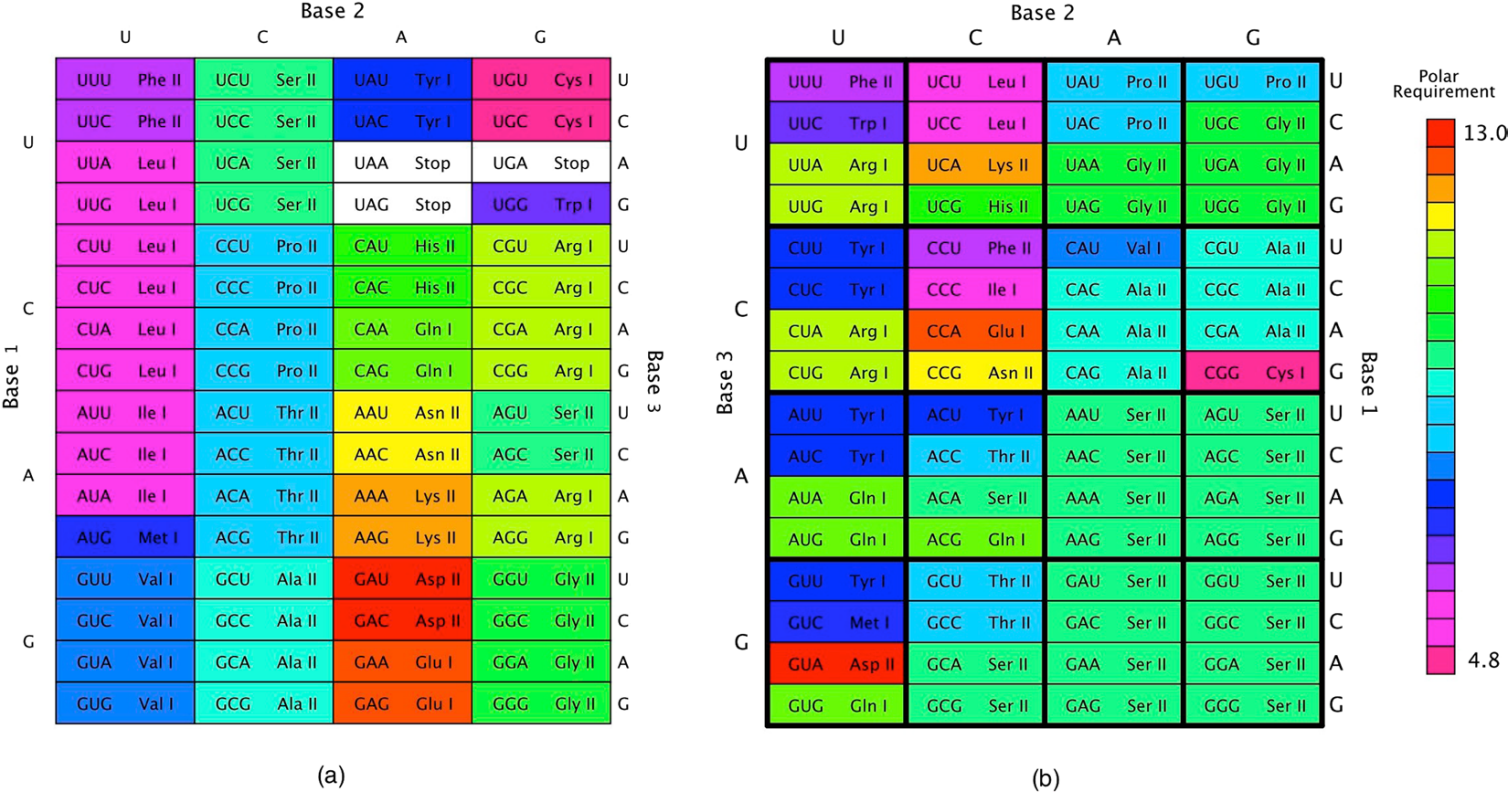
Assignments of the 64 codons of the genetic code. The bases of the codon table are arranged according to their specific error robustness: least (top), middle (left), and most robust (right). An amino acid’s slot is coloured according to its polar requirement to illustrate chemical similarity. Its aminoacyl-tRNA synthetase class is I or II. (**a**) The highly ordered standard genetic code. Stop codon slots are coloured white. (**b**) A highly robust artificial code emerging from the iterated learning model. Stop codons were not included in the model.

Explaining the origins of life remains one of the biggest challenges of science, and one essential aspect of this challenge is to explain the origin of the standard genetic code. Any theory of why the standard genetic code is the way it is and how it came to be must address three key facts: (1) the code’s *regularity* as expressed in non-random amino acid assignments, (2) its *optimality* as expressed in its robustness against errors in translation from code sequences to proteins and in replication of genetic material, and (3) its near *universality* across extant biological systems. The standard genetic code’s regularity and consequent optimality can be seen in the highly ordered arrangement of the codon table (Fig. 1a). Yet a current review of over 50 years of research concluded that despite much progress in this field we do not seem to be much closer to such a theory^1^.

The theory that the standard genetic code was shaped by natural selection to minimize adverse effects of mutation and/or mistranslation is most widely accepted because it can explain the code’s regularity and optimality^2^. Nevertheless, this theory takes vertical descent as its starting point, and thus to some extent assumes as given that which it sets out to explain. Moreover, this theory sits uneasily with the finding that standard genetic code is actually not that optimal, at least according to some measures, and that better codes can be found when specifically selected for robustness^3^. This sub-optimality has been interpreted as a combination of adaptation and frozen accident, whereby selection was constrained by initial conditions and subsequently by deleterious consequences of code changes.

Another possibility is suggested by the existence of simplified genetic codes^4^: if codes started with few amino acids, then optimality could be an emergent consequence of code expansion from early simple ones to the standard genetic code, whereby new amino acids were assigned to codons that had previously encoded chemically similar amino acids^5^. This theory appeals to mutation, and in some cases selection^6^, to account for the inclusion of new amino acids, and therefore still assigns a key role to vertical descent, but some of its versions are neutral with respect to the role of selection^7^.

Crick’s frozen accident hypothesis^5^ also helps to explain universality because all extant species share a last universal common ancestor, and thus inherited its genetic code. However, this last universal common ancestor must have already been a highly optimised organism, which leaves us with a circular problem that is somewhat similar to Eigen’s paradox: it is difficult to conceive of the evolutionary origins of its complex translation system before there were a number of functional proteins capable of maintaining the integrity of the system to minimize the translational error along with functional RNAs, but such proteins could not have evolved without a high-fidelity translation system^8^.

Woese proposed that this bootstrapping problem could have been overcome by shifting the problem from the translation system to the genetic code^9^. For although the earliest forms of life did not yet have the proteins to improve their translation system, they could have done something tantamount to this by adjusting their genetic code so as to mitigate the deleterious effects of errors. He conjectured that early evolution was dominated by horizontal exchange of cellular components among loosely organized protocells (“progenotes”), rather than by vertical transmission of genetic material^10,11^, and that lineages of individuals did not exist until after the emergence of the last universal common ancestor (the “Darwinian threshold”)^8,12^. In addition, Woese argued that this pre-Darwinian mode of communal evolution would have enhanced the evolvability of protocells by increasing the availability and spread of genetic novelty^10^. This would additionally have generated selection pressure for universality because mutual genetic intelligibility was necessary for receivers to take advantage of donors’ innovations^13–15^. Woese proposed this scenario of communal evolution partly to explain how the last universal common ancestor became so complex so rapidly^12^, a problem that remains pertinent given evidence of life’s rapid emergence^16,17^. Crucial outstanding problems with this proposal are clarifying the agency of selection in communal evolution^18^, and verifying whether it provides a basis for the key properties of the codon assignments of the standard genetic code^19^. We set out to respond to these open problems by implementing an agent-based computer model of communal evolution.

Previous computer models have confirmed that adding horizontal gene transfer to the evolution of a population of genetic codes selected for error robustness has the effect of facilitating convergence on optimal and universal codes compared to vertical descent alone^20–22^. This is a Lamarckian mode of evolution with an explicit role for lifetime reorganization of the recipient cell^10^, which is assumed to detune its code for purposes of recognizing alien genes encoded by the donor’s table, thereby forming a mixed code^20^. Nevertheless, all of these models continue to take vertical descent as their starting point and therefore leave it unresolved if a purely Woesian mode of evolution, that is, if a process of communal evolution of traits *without* vertical descent, could by itself account for the key properties of the standard genetic code.

Yet Woese’s notion of communal evolution is not as mysterious as it might initially seem because the general process is already familiar from language evolution. One popular theory is that linguistic structure is an emergent outcome of the pressures of intergenerational transmission based on sparse sampling available to individual learners, and that this structure shaped the fitness landscape for the biological evolution of the language faculty^23^. The iterated learning model that is commonly used to support this theory^24^ therefore served as our inspiration for simulating the communal evolution of the genetic code. A virtue of the iterated learning model is that it enables us to explain the origins of a complex symbol system without having to posit a similarly complex internal symbol system. Specifically, in the case of language evolution we can explain the origins of linguistic structure without having to assume the existence of an innate universal grammar that was evolved by natural selection.

The general similarities between the appearance of human language and the appearance of the genetic code, which both involved the creation of new forms of translation, have already been widely noted^25^, including by Woese^10^. Here we make use of this analogy to propose an iterated learning model of the genetic code. In particular, we show that a suitably adapted iterated learning model may similarly explain the origins of the standard genetic code, without forcing us to assume that this required the kind of genetic system found after the last universal common ancestor fully capable of translation and replication. Our efforts can be seen as part of a broader shift away from an overly restrictive focus on internal processes toward a deeper recognition of the essential role of interaction dynamics for explaining the complexities of mind and life^26,27^.

**Figure 2:**
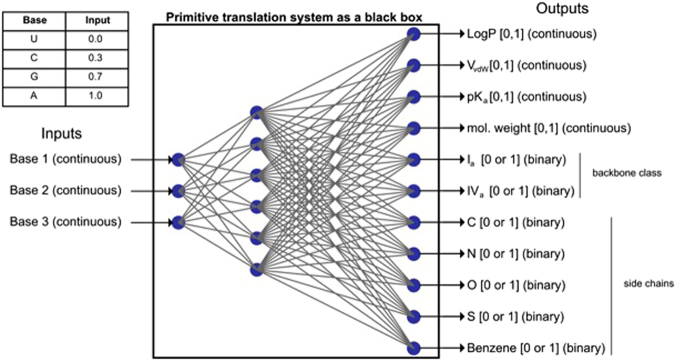
‘Black box’ of a protocell’s primitive translation system. For simplicity, and following previous work on the iterated learning model, we used a fully interconnected feed-forward multi-layer perceptron network to model the translational mapping from a codon to its corresponding amino acid. There are three input nodes, one for each of a codon’s bases. The order of base positions is arbitrary and interchangeable (no third base ‘wobble’). There are six hidden nodes. Output is an 11-dimensional vector that specifies an amino acid in terms of properties by which it can be uniquely distinguished in chemical space.

In accordance with Woese^10^, we assume that the protocells subject to communal evolution had not yet completed the genotype and phenotype relationship of modern cells (Woese and Fox’s “progenotes”^28^). We do not pretend to know how such a protocell was internally organized. In his later work Woese envisioned them as “supramolecular aggregates”^10^, consisting of a self-sustaining metabolic network that was incorporated into a modular higher-order architecture formed around nucleic acid componentry. For the purposes of our model we ignore the metabolic network, and we treat the primitive translation system as a ‘black box’ system that is capable of nonlinearly mapping from an input in nucleotide space (a codon) to an output in chemical space (an amino acid). For simplicity, and following previous work with iterated learning models^29^, this ‘black box’ was implemented as a multilayer perceptron network capable of backpropagation learning (Fig. 2). This particular implementation is admittedly not a realistic architecture for protocells, but it is at least within the possibilities of chemical networks in principle^30^.

Woese argued that the componentry of protocells was sufficiently simple and modular such that most of it could have been exchanged between spatially close protocells, and thereby further altered and refined in a communal way. Given that Woese assumed that the gene-trait relationship was not yet in place at this early stage of communal evolution, we therefore conceived of this process of horizontal transfer not as a form of gene transfer, but rather as the transfer of all kinds of sufficiently modular components between protocells, including parts of the sender’s translation system that could be interpreted by the recipient as fragments of its genetic code.

For simplicity, we assume that the three-base structure of the codon was already in existence; it could have emerged as the most stable configuration of the translocation dynamics of the primitive translation system^31^. We did not include mRNA with consecutive codons, although we can imagine polymerization based on spatial proximity^32^ and template properties of surfaces^33^. The resulting short oligonucleotides could have already conferred selective advantages, for example by playing regulatory functions^34^, or by playing the role of a catalyst for nonenzymatic replication of RNA^35^.

All 20 amino acids of the standard genetic code are assumed to be available for encoding. We do not propose that all 20 were used in early genetic codes, in particular given that several depend on complex biosynthetic pathways^36,37^, but by making all of them potentially available for encoding in our model we could study the relative probabilities of their fixation in the emerging codes. No stop codons were included. This is in line with previous work that assumed that the earliest translation mechanism did not require special start and stop codons^6^.

**Figure 3:**
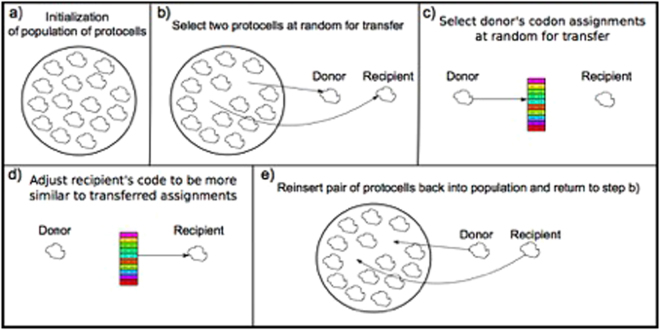
A model of communal evolution of the genetic code. (**a**) A small group of protocells is initialized such that their ‘black box’ primitive translation systems encode random genetic codes consisting of few amino acids. Then the ‘iterative learning’ cycle begins. (**b**) Two protocells are randomly selected for horizontal transfer of a fragment of the donor’s genetic code to the recipient. (**c**) A small subset of codon assignments is randomly chosen and transferred; occasionally, codon assignment inaccuracies can occur in the transferred components. (**d**) The recipient adjusts its genetic code to be more like the donor’s code according to the received assignments. (**e**) The process of horizontal transfer is completed. Then the cycle starts again by going back to (**b**).

We start a run with a small group of protocells (see Methods section for details) that are assumed to be in close spatial proximity, perhaps by being contained in a small hydrothermal pool^38^ or in a gel phase^39^. Every protocell’s ‘black box’ translation system is initialized with random connection strengths such that only a small number of amino acids are initially encoded (i.e. the initial translation system lacks discriminatory capacity and this results in low expressivity). We then repeatedly select a random donor and receiver protocell for horizontal transfer (Fig. 3). This could have involved pairs of tRNAs with their cognate amino acid tRNA synthetase or, more simply, a single RNA strand in which tRNA and aminoacylation ribozyme were fused^40^. Effectively, each time a small subset of randomly selected distinct codon assignments are transferred (we arbitrarily chose 10). Probabilities of transfers are biased according to how frequently the amino acids are used in contemporary cells^41^. Following previous work^20,21^, we assume that the receiver then uses these translational components to make its code more similar to the donor’s code. Given the limited capacities of the primitive translation system, it can only adjust its code imperfectly and we assume this would involve an overall alteration of the receiver’s code. We do not know the possible biochemical basis for this lifetime reorganisation of the receiver’s translation system, and we treat it as part of the black box.

The transferred code assignments do not necessarily perfectly reflect the donor’s code because occasionally codon assignments to other amino acids are possible, even if they have not yet been specifically encoded in the donor’s codon table. This source of novel amino acid assignments could have derived from the lack of specificity of the precursor tRNAs. In such a case, we follow Crick’s^5^ conjecture that similar amino acids will tend to end up with similar codons.

To be clear, the model only consists of iterations of a certain type of protocellular interaction: a sender’s horizontal transfer of fragments of its code followed by the receiver’s corresponding code adjustments that increase their similarity. There is no vertical descent in the model: protocells do not have their fitness evaluated; they are not selected; and they are not replaced by offspring with mutations. Genetic codes can emerge on the basis of this iterated protocellular interaction alone.

## Results

After tens of thousands of iterations of horizontal transfer, artificial genetic codes start to emerge that exhibit the key features of the standard genetic code.

### Optimality

**Figure 4:**
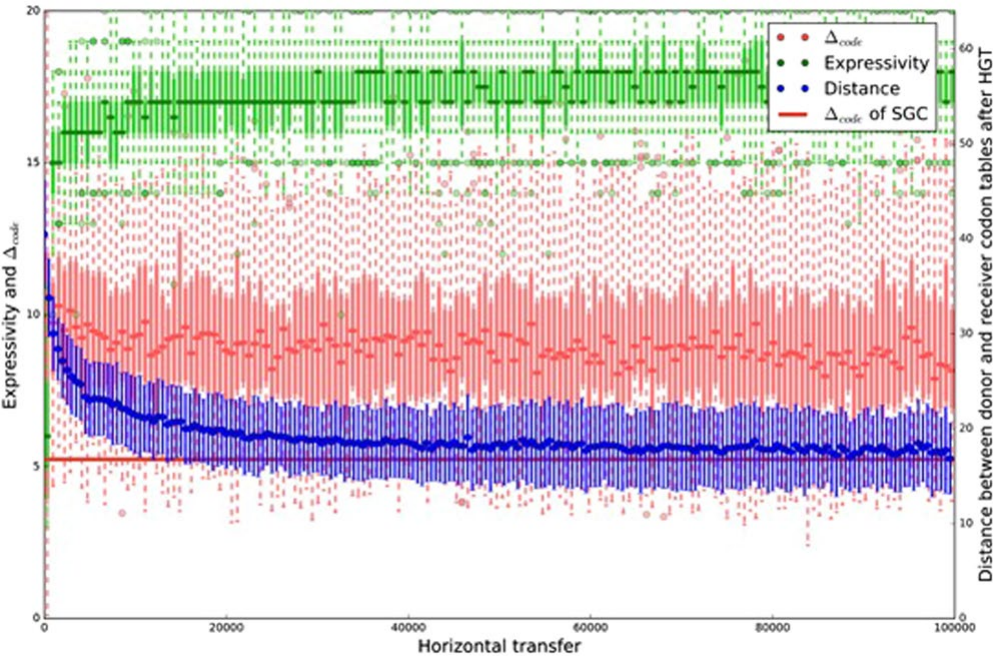
Emergence of artificial genetic codes. Results are averaged from 50 runs and plotted in intervals of 500 transfers. Expressivity counts the receiver’s encoded amino acids after a transfer (range [1, 20]), plotted as a box plot where the dark green bar represents the overall mean, the lighter green bar represents lower and upper quartiles, dotted lines represent minimum and maximum non-outliers, and circles represent outliers. Δ_code_ represents optimality as the code’s robustness to single nucleotide changes (red box plot). The standard genetic code (SGC) has an expressivity of 20, the number of amino acids encoded in the code. The Δ_code_ of SGC’s codons (excluding stop codons) is 5.24 (red line). The most robust artificial code with the same expressivity as the SGC has a Δ_code_ of 4.17 (see Fig. 1b for details). Universality is measured as the average distance between all codes in a group of protocells, where distance is calculated as the number of different codon assignments (range [0, 64]). We plot the overall mean distance and its standard deviation, with the final average of 16.86 different assignments being the smallest overall average encountered for the duration of these runs.

Single nucleotide changes to codons tend to result in assignments to the same or to a chemically similar amino acid (e.g. using the polar requirement scale, see Fig. 1b). The optimality of the most robust codes that encode all 20 amino acids are within range of the standard genetic code, but do not significantly go beyond it (Fig. 4). Communal evolution therefore does not have the problem associated with infinite population models of Darwinian selection of error robustness, which can give rise to artificial codes that are much more error robust compared to the standard code. In this sense the results are more similar to those obtained by finite population models of Darwinian code selection^42^.

### Universality

We measured universality as the decreasing number of different assignments. The genetic codes used by a group of protocells tend to become more similar to each other over time (Fig. 4). This process of code convergence at first proceeds rapidly but then continues more slowly than expected. After 100,000 iterations the average distance between codon tables was still decreasing, and at this point it is therefore not clear how much further the remaining code diversity could be reduced. At the end of the current simulation runs artificial code similarity never reached the near universality of the standard genetic code, for which differences in codon assignments are extremely rare, even when considering that they are much more frequent than previously expected^43^. Nevertheless, it seems reasonable that substantial variations in the code would have been frequent before the last universal common ancestor, after which changes to the code became costly and difficult^44^. We will return to this point in the discussion.

### Regularity

**Figure 5:**
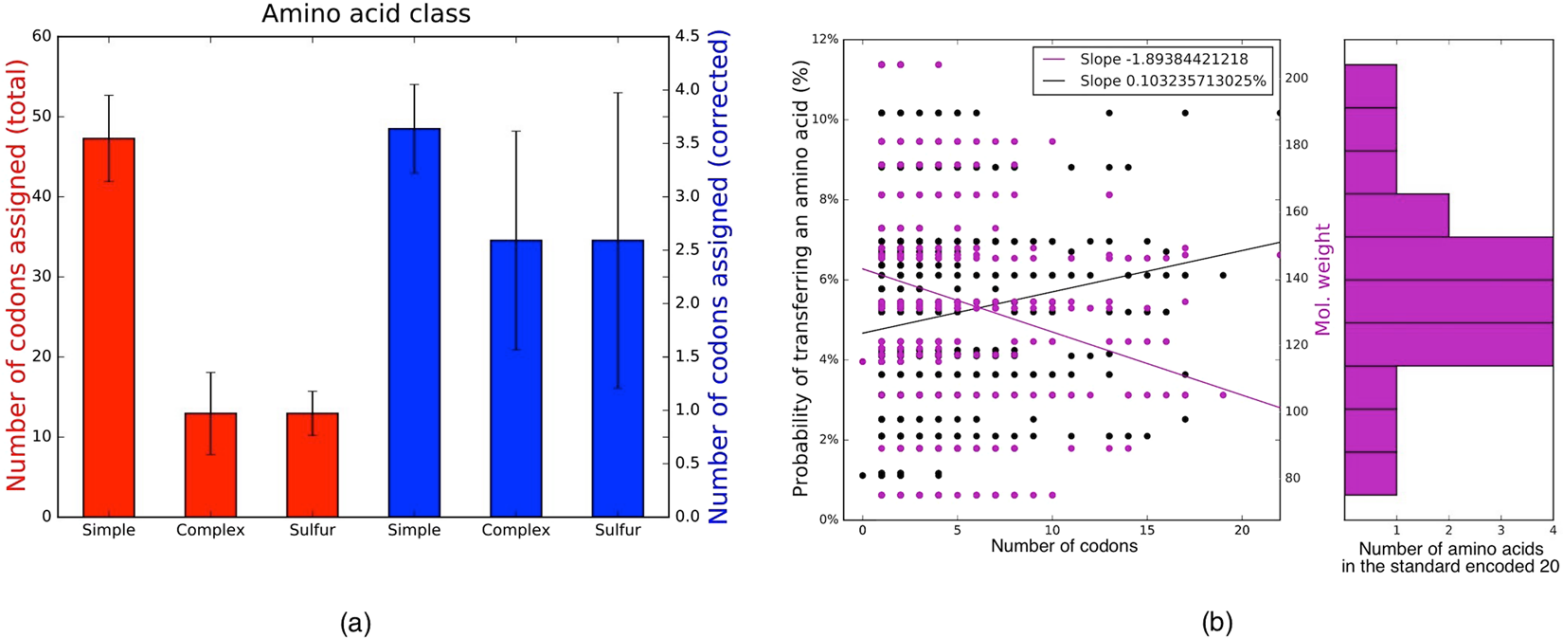
Regularities of the artificial genetic codes. We analysed the average properties of the 50 most optimal artificial genetic codes, one from each of 50 the independent runs. (**a**) Like the standard genetic code, the class of simple amino acids has more assignments than the complex and sulfur classes (red). This may partly result from the fact that the simple class is more frequent among the 20 encoded amino acids, but this tendency remains even if we correct for the unequal distribution of classes (blue). (**b**) Like the standard genetic code, there is a positive correlation between an amino acid’s frequency in proteins, modelled in terms of probability of amino acid transfer, and number of assignments (black). And there is also a negative correlation between its molecular weight and number of assignments (purple). Again, this may partly result from the fact that lighter amino acids are more frequent among the 20 encoded amino acids.

The artificial codes exhibited several regularities that are also known from the standard genetic code, and which seem to require a special explanation^45^. First, there is a negative relationship between amino acid complexity and number of codon assignments^8^, such that simple ones have more assignments than complex ones and ones containing sulfur (Fig. 5a). Second, there is also a negative relationship between amino acid molecular weight and number of codon assignments^46^, such that lighter ones have more assignments than heavy ones. Third, there is a positive relationship between an amino acid’s frequency in extant organisms (and hence its probability of transfer) and its number of codon assignments^47^, such that more frequently transferred amino acids have more assignments (Fig. 5b). This third regularity may help to explain the first two, given that more complex and heavier amino acids are thermodynamically more costly and therefore can be expected to be relatively less frequent^48^. Indeed, simpler and lighter amino acids are more frequent among the 20 encoded amino acids, so these effects are expected to persist even if we do not bias transfer probabilities according to their relative frequency in proteins.

However, there are also some missing regularities. For example, the standard genetic code exhibits a link between the second codon letter and chemical properties of the encoded amino acid, such that a *U* corresponds to hydrophobic amino acids^49^. Another example is that the standard genetic code is characterized by a relationship between the second codon position and the encoded amino acid’s class of aminoacyl-tRNA synthetase^50^. The artificial codes tend toward an ordered arrangement of synthetase classes, but there tend to be exceptions (see, e.g., Fig. 1b).

## Discussion

The model’s results significantly lower the bar for what could be in principle required for the optimality, universality, and regularity of the standard genetic code to appear. The model serves as a first formal proof of concept that it is not necessary for theories of the origins of the standard genetic code to assume an essential role for vertical transmission with a high-fidelity translation and replication system. In addition, given that the model does not include any role for gene sequences coding for protein structures, this supports the possibility that the existing regularities in the standard genetic code reflect more basic amino acid properties^8^. Given that the model depends on repeated horizontal interactions between protocells, it would favour a scenario for the origin of life that can ensure close spatial proximity of small populations, like a hydrothermal pool^38,51^.

A key result of the model is that more frequently transferred amino acids tend to have more codons assigned to them, which results in several regularities known from the standard genetic code. How this happens in the model is an interesting question that deserves more systematic investigation. We hypothesize that it has do with the fact that protocells are required to incorporate these amino acids into their codes more often and yet, given the initial diversity of codes, have to accommodate the diversity of codons assigned to them. The combination of these two factors may result in these amino acids appearing with different codons assigned to them.

The absence of letter-specific regularities in the artificial codes was a surprise, especially given that in the context of language evolution the iterated learning model is known to give rise to combinatorial structures such that certain subparts of letter strings come to refer to specific traits of the referents. This lack of letter-specific coding in our artificial codes may be partly a consequence of the fact that, in contrast to the standard genetic code, the emerging codes could make equal use of all three letters of a codon, resulting in codes that distributed encoded properties more evenly across all letters. Given the possibility of equal use, our model does not give rise to effects related to the “third base wobble,” which otherwise could have provided another source of regularity. Nevertheless, given that compositionality is a common finding in linguistic iterated learning models, it is expected that this kind of regularity should be within the realm of possibility of our model if the right conditions for their emergence are found.

Moreover, the missing regularity of aminoacyl-tRNA-synthetase class assignments to a specific letter may be considered as a direct consequence of the first: if Woese and colleagues^52^ were correct in concluding that it is unlikely that the aminoacyl-tRNA synthetases played any specific role in shaping the evolution of the genetic code, then the ordered arrangements of their classes exhibited by the standard genetic code may have been an indirect consequence of adapting to pre-existing regularities in codon assignments, rather than of directly shaping those regularities in the first place.

Finally, the model suggests that another mode of evolution was operative at the origins of life: not only was Darwinian evolution (vertical transmission of genes) arguably preceded by Lamarckian evolution (vertical transmission of genes and acquired traits)^11^, the latter was possibly preceded by Woesian evolution (horizontal transmission of acquired traits). This model shows how the protocells themselves could serve as their own agents of selection in Woesian evolution, given that only genetic codes that can be sufficiently assimilated by the recipients of the code fragments can be subsequently horizontally transferred to the next protocell. It further suggests that this communal evolution could give rise to key properties of the genetic code because the need for the codes to be horizontally transferable puts pressure on the codes to become more regular and redundant, otherwise they are likely to disappear.

Nevertheless, despite long-term convergence of the codes, their diversity remained notably higher than in previous models of code evolution that had included horizontal transfer, which in hindsight may not be entirely unexpected given the complete lack of competition between protocells in our model. Code diversity is influenced by at least two factors: increasing the number of codons sent per transfer has a homogenizing effect, while more individuals in the population means more diversity. It is therefore likely the case that the convergence on a more universal code found in previous models crucially depends on their inclusion of an explicit selection pressure, whereby protocells have competing fitness with respect to error robustness. In our model, on the other hand, there is no selection pressure and therefore the only pressure driving code convergence is each protocell’s adjustments leading to increasing code similarity between sender and receiver.

Our results therefore suggest a complementary scenario to the proposal by Goldenfeld *et al*.^22^, according to which a communal mode of evolution involving vertical descent would have converged on a universal code before or during the transition to mainly vertical evolution. Our model suggests that before the appearance of vertical descent there might have been a diversity of codes with different extents of overlap, and at some point one or several of them had become sufficiently robust to errors such that vertical descent could become a part of their dominant mode of evolution in addition to horizontal transfers. This allowed them to subsequently outcompete the other codes by also vertically accumulating innovations until only one dominant code remained, in a mode of Lamarckian evolution similar to Goldenfeld *et al*.’s proposal.

## Methods

### Multi-layer perceptron system

A multi-layer perceptron serves as the ‘black box’ primitive translation system. It has three input nodes, six hidden nodes, and 11 output nodes. The input of the system is a codon triplet, i.e. there is one input node per codon base position. Input is in the range [0, 1]. The output is a point in the 11-dimensional chemical space, which defines a particular amino acid (see below for details). We represented the fact that the four letters of the standard genetic code can be divided into two classes of nucleobases, i.e. pyrimidine derivatives (U, C) and purine derivatives (A, G), by distributing them unevenly in input space (U = 0, C = 0.3, A = 0.7, G = 1). The order of the three input bases is arbitrary and interchangeable (i.e. the model does not include an uneven distribution of assignment uncertainty due to a third base ‘wobble’). There is no codon ambiguity; each codon maps uniquely to one amino acid. Six hidden nodes were sufficient for the system to be able to map 64 codons to 20 amino acids. Although we did not investigate the role of the number of hidden nodes systematically, exploratory tests revealed that a significantly reduced internal complexity made it more difficult to acquire a full expressivity of 20 encoded amino acids, whereas a significantly increased internal complexity facilitated this process but at the cost of making the model less computationally tractable. Input, hidden, and output layers are fully connected in a feed-forward manner such that each node in one layer connects with a weight *w*_*ij*_ to every node in the following layer. There is also a bias node that is connected with a weight *w*_*bj*_ to each node in every layer (not shown in Fig. 2). Except for the input nodes, each node in a multi-layer perceptron has a nonlinear activation function, typically a sigmoid, that determines its output. Because we wanted output to be in the range [0, 1] we used the logistic function described by *y*_*i*_(*v*_*i*_) = (1 * *e*^−*v*^_*i*_)^−1^, where *y*_*i*_ is the output of the *i*^th^ node and *v*_*i*_ is the weighted sum of its incoming connections. At the start of a simulation run each protocell’s multi-layer perceptron has all of its connection weights initialized to a random value drawn from a uniform distribution with a mean of 0 and standard deviation of 0.1.

### Chemical space

The output of the ‘black box’ primitive translation system is an amino acid specified in terms of its properties in chemical space. Following previous literature^53,54^, we included three basic properties, namely amino acid *size* (*V*_*vdW*_, van der Waals volume, measure of the total volume of the molecule enclosed by the van der Waals surface), *charge* (*pK*_*a*_, which is the negative log of the acid dissociation constant for an amino acid side chain), and *hydrophobicity* (log *P*, which is the partition coefficient, a measure of the distribution of the molecule between two solvents). The values for these properties are the same as those used by Ilardo *et al*.^54^. We also included *molecular weight* as another defining amino acid property because it has been observed to have a non-random distribution within the codon-amino acid matrix of the standard genetic code^46^. The values for each of these basic properties were normalized to the range [0, 1]. We also included more complex amino acid properties, including five *side chain properties* (C, N, O, S, and benzene) and two *backbone types* (Ia and IVa)^55^, each specified as either present (1) or absent (0). All of this data can be found in the Supplementary Information online. These choices resulted in an 11-dimensional chemical space in which all of the 20 encoded amino acids of the standard genetic code could be uniquely specified. This restriction to the 20 standard amino acids meant that codes were prevented from encoding other amino acids and from increasing their expressivity beyond 20 amino acids. Future work could lift this restriction to investigate what kind of codes would emerge when confronted with the full space of possible amino acids.

### Backpropagation learning

We treat the process by which the recipient adjusts its code to the donor’s code, which would improve recognition of donated genetic material, as another ‘black box’. For simplicity, and following a long tradition of iterated learning models^56^, we utilized a standard supervised learning technique called backpropagation to modify an multi-layer perceptron’s input-output mapping. The error of an output node *j* for the *n*^th^ training sample is given by *e*_*j*_(*n*) = *d*_*j*_(*n*) − *y*_*j*_(*n*), where *d*_*j*_ is the node’s target output value and *y*_*j*_ is its actual output value. Network weights are adjusted so as to minimize the sum of output errors, which is given by Equation (1):

1 $$\varepsilon (n)=\frac{1}{2}\sum _{j}{e}_{j}^{2}(n)$$

The change of each weight can then be calculated using the standard gradient descent method, as described in Equation (2):

2 $${\rm{\Delta }}{\omega }_{ji}(n)=-\eta \frac{\delta \varepsilon (n)}{\delta {\upsilon }_{j}}{y}_{i}(n)$$

where *y*_*i*_ is the output of the previous neuron and *η* is the learning rate, which we set to 0.1. Future work could model this whole ‘black box’ system in a chemical network, whose capacities for learning are beginning to be better understood^30,57–59^.

### Iterated learning model

The iterated learning model of language evolution^24^ requires the following elements: (1) a meaning space, (2) a signal space, (3) one or more language *learning* agents, (4) and one or more language *teaching* agents. For the purpose of studying genetic code evolution we re-interpreted these requirements as follows: (1) a chemical space of amino acids, (2) a set of codons, (3) one or more recipient protocells, and (4) one or more donor protocells. A donor transfers a subset of signal-meaning pairs, which we can imagine to consist of proto-tRNAs fused with their own aminoacylation enzymes, to a receiver (see below for details). The receiver must then adjust its code to be more like the donor’s code based on this sparse sample of the donor’s code. Woese does not go into the details of this assimilation process other than noting that the receivers, who adjust their codes so as to be better able to take advantage of foreign genetic material, dominate the dynamic of evolution via horizontal transfers.

After a number of iterations the codes will start to exhibit order that makes them more learnable, given that donor code properties that are difficult to imitate by a receiver are unlikely to be then transferred on by that receiver to another receiver. In the case of language evolution best results are obtained by ensuring a one-way chain of horizontal transfers from mature to immature agents, where the former are defined as having had more opportunity for learning than the latter, because this arrangement avoids interference caused by receiving disorganized languages from immature agents. Although such an iterated unidirectional sequence may be plausible for language evolution, where children are more likely to learn from adults than vice versa, it is unrealistic for genetic code evolution. Accordingly, we opted for a parallel scenario involving a small group of protocells, which we can imagine persisting in close spatial proximity, for example by being contained in a hydrogel^39^. In line with another parallel population-based iterated learning model^60^, we set the total community size to *N* = 16.

### Horizontal transfer

Pairs of protocells were randomly selected for horizontal transfer in an iterative manner. We did not place any restrictions on who could be the donor and who could be the receiver. We assume that the donor transfers a small amount of its translational components, reflecting a random subset of its codon table, to the receiver. Specifically, we randomly selected 10 codon assignments to distinct amino assignments. Each amino acid has a relative probability of being transferred that is inversely related to its thermodynamic cost because less costly amino acids were likely more abundant^48^, i.e. simpler ones are more likely than complex or sulfur ones. Since the relative frequency of amino acids during the early phase of evolution is unknown, we used an estimate of the cellular relative amino acid abundance (cRAAA) of modern organisms^41^. To create signal-meaning pairs, for each selected amino acid to be transferred we had to determine its codon assignment according to the donor’s code. In line with previous work on iterated learning models we employed an obverter function^29^: we evaluate which codon input to the donor’s network produces the output in chemical space that most closely resembles the target amino acid. Occasionally, an error would occur whereby a codon would be assigned not to the amino acid specified by the donor’s code, but to another amino acid nearby in chemical space. This serves as the driving force for increasing amino acid diversity in the population.

The receiver’s primitive translation system is presented with the 10 codon-amino acid pairs in a random order. After each input codon presentation the output produced by the receiver’s multi-layer perceptron is compared to the expected (i.e. donor’s) output and the connection weights are adjusted according to the backpropagation algorithm. The network is trained in this manner 500 times per horizontal transfer. A simulation run typically consists of several tens of thousands of transfers. We arbitrarily stopped the runs after 100,000 transfers.

### Measures

*Expressivity* counts the number of encoded amino acids in the receiver’s code after adjusting its code to be better able at recognizing the donor’s code. The *Δ*_*code*_ measures the receiver’s code optimality, calculated by iterating through all of the codons assigned to amino acids and performing all possible single nucleotide changes, summing the average distance between these neighbouring codons’ amino acids in chemical space (measured by their mean square difference in polar requirement), and then averaging over all codons (for the model this includes all 64 codons, but for the standard genetic code it only includes 61 because the three stop codons are excluded). We measured code *universality* as the average distance between all protocells in terms of the number of differences in amino acid assignments.

## Electronic supplementary material

Supplementary Information
