# Supplementary material for: Horizontal transfer of code fragments between protocells can explain the origins of the genetic code without vertical descent
Source: Sci Rep. 2018 Feb 23;8:3532. doi: 10.1038/s41598-018-21973-y (PMC5824800; doi:10.1038/s41598-018-21973-y)
Supplement: Supplementary file 1 — Supplementary Information [file 41598_2018_21973_MOESM1_ESM.docx]

**Horizontal transfer of code fragments between protocells can explain the origins of the genetic code without vertical descent**

Tom Froese, Jorge I. Campos, Kosuke Fujishima, Daisuke Kiga,

and Nathaniel Virgo

| **Original sources of data:** | | |
| --- | --- | --- |
| V_vdW, pka, logP |  | Ilardo, M., Meringer, M., Freeland, S. J., Rasulev, B. & Cleaves II, H. J. Extraordinarily adaptive properties of the genetically encoded amino acids. *Scientific Report*s **5**, doi:10.1038/srep09414 (2015) |
| mol. Weight |  | Hasegawa, M. & Miyata, T. On the antisymmetry of the amino acid code table. *Origins of Life* **10**, 265-270 (1980) |
| Side chains and backbone class | | Meringer, M., Cleaves II, H. J. & Freeland, S. J. Beyond terrestrial biology: Charting the chemical universe of α‑amino acid structures. *Journal of Chemical Information and Modeling* **53**, 2851-2862 (2013) |
| cRAAA (d) |  | Moura, A., Savageau, M. A. & Alves, R. Relative amino acid composition signatures of organisms and environments. *PloS ONE* **8**, e77319, doi:10.1371/journal.pone.0077319 (2013) |
| Class |  | Smith, E. & Morowitz, H. *The Origin and Nature of Life on Earth: The Emergence of the Fourth Geosphere*. (Cambridge University Press, 2016) |
| Polar requirement |  | Lenstra, R. The graph, geometry and symmetries of the genetic code with Hamming metric. *Symmetry* **7**, 1211-1260 (2015) |
